# Pathophysiological Models of Hypersomnolence Associated With Depression

**DOI:** 10.1016/j.bpsgos.2024.100445

**Published:** 2024-12-26

**Authors:** Christophe Moderie, Diane B. Boivin

**Affiliations:** Department of Psychiatry, McGill University, Montreal, Quebec, Canada; Centre for Study and Treatment of Circadian Rhythms, Douglas Mental Health University Institute, McGill University, Montreal, Quebec, Canada

**Keywords:** Circadian rhythms, Excessive daytime sleepiness, Hypersomnolence, Mood disorders, Sleep

## Abstract

Up to 25% of patients with depression experience hypersomnolence (e.g., excessive daytime sleepiness, hypersomnia, and/or sleep inertia), which is associated with treatment resistance, overall poorer outcomes, and safety concerns while driving. Hypersomnolence can result from various sleep/neurological disorders or side effects from medication but is often medically unexplained in depression. In this review, we aimed to summarize the different pathophysiological models of hypersomnolence in depression to discuss their impact on nosology and to foster the development of better tailored diagnostics and treatments. We identified several potential mechanisms underlying hypersomnolence including a daytime hypoactivity of dopaminergic and noradrenergic systems, nighttime GABA (gamma-aminobutyric acid) hypoactivation, hypoperfusion, and hypoconnectivity in the medial prefrontal cortex, as well as a longer circadian period and light hyposensitivity. In some patients with depression, nighttime hyperarousal can fragment sleep and result in a complaint of excessive daytime sleepiness, thus mimicking hypersomnolence. Others might adopt maladaptive behaviors such as spending excessive time in bed, a term coined clinophilia. Objective markers of hypersomnolence, such as ambulatory ad libitum polysomnography may facilitate distinguishing between conditions that mimic hypersomnolence. Our review identified several clinical targets for hypersomnolence in depression. Low-sodium oxybate, which is approved for idiopathic hypersomnia, needs additional study in patients with depression. Neuromodulation that targets prefrontal cortex anomalies should be systematically explored, while tailored light therapy protocols may mitigate light hyposensitivity. Additionally, cognitive behavioral therapy for hypersomnolence is being developed as a nonpharmacological adjunct to these treatments.

## Plain Language Summary

Many people with depression experience hypersomnolence, which encompasses excessive daytime sleepiness and prolonged sleep duration. Hypersomnolence is associated with safety issues, such as driving concerns, and makes depression more difficult to treat. The causes of hypersomnolence in depression are unknown. In this review of the literature, we synthesize potential causes, including reduced daytime activity in brain systems that regulate alertness, nighttime sleep disruptions, altered circadian rhythms, and behaviors like spending extended time in bed. Understanding these mechanisms may support the development of targeted treatments, such as medications, specific light therapy protocols, and customized cognitive behavioral approaches, to better manage hypersomnolence in depression.

Sleep and mood disorders are interconnected (1), and although insomnia is commonly linked to depression, up to 25% of patients with major depressive disorder (MDD) report hypersomnia (2, 3, 4). Hypersomnia, together with excessive daytime sleepiness (EDS) and sleep inertia, define the broader concept of hypersomnolence. Hypersomnia refers to a prolonged sleep duration of over 9 to 12 hours based on various definitions (5,6). EDS is the inability to maintain wakefulness or alertness throughout the major waking episodes, which results in periods of irrepressible need for sleep or unintended lapses into drowsiness or sleep (4,6). Sleep inertia describes difficulties with sleep-to-wake transitions, often involving sleep reentries, reduced vigilance, and impaired cognitive performance lasting minutes to hours. Sleep drunkenness, an extreme form of sleep inertia, includes clumsiness and confusion upon awakening (7). Those symptoms can severely impact functioning, quality of life, and safety in situations such as driving or operating equipment. In depression, hypersomnolence is associated with treatment resistance and poorer outcomes (8). Hypersomnolence occurs in various sleep and neurological disorders, such as narcolepsy and Parkinson’s disease. A pathophysiological model was established for disorders such as narcolepsy, but the etiology remains unknown in depression. This results in limited treatment options and few specific guidelines (9, 10, 11).

A challenge in studying hypersomnolence is refining nosology to better characterize these phenomena. Improved terminology is crucial to accurately describe symptoms and thereby potentially aid the understanding of pathophysiological mechanisms. Hypersomnolence can be a symptom of depression according to DSM-5-TR, but if excessive, it could be diagnosed separately as a hypersomnolence disorder (5). Hypersomnolence disorder was introduced in DSM-5 based exclusively on patient-reported symptoms (5). The International Classification of Sleep Disorders, Third Edition, Text Revision (ICSD-3-TR) defines another entity of unexplained hypersomnolence called idiopathic hypersomnia (IH), which has more stringent criteria and requires objective testing with the Multiple Sleep Latency Test (MSLT). The MSLT, a full-day test with 5 naps, is the current gold standard to assess EDS (12). Pathological sleepiness is defined as a mean sleep latency ≤8 minutes across naps (12). The MSLT follows a night of polysomnography (PSG) to assess sleep efficiency, duration, and other sleep disorders. Diagnostic considerations and differences in sleep medicine and psychiatry nosologies regarding hypersomnolence are further elaborated in our recent review (13).

The heterogeneity of depression complicates studies on its link to hypersomnolence. MDD has multiple specifiers, including with atypical features, which has hypersomnia as a diagnostic criterion (5). MDD can also be with seasonal pattern (seasonal affective depression [SAD]), for which hypersomnia is typically described but is not included in the diagnostic criteria (5). Bipolar disorders, which include type 1 (history of manic episodes) and type 2 (hypomanic and depressive episodes), also often present with hypersomnolence (5). In addition, depressive episodes in bipolar disorder can present with the same features as MDD. The ICSD-3-TR also lists psychiatric disorders that can co-occur with depression and hypersomnolence, including somatoform, adjustment, and personality disorders (14). Because studies often lack detailed clinical descriptions, we used depression inclusively, specifying populations when possible.

In this narrative review, we aimed to synthesize evidence on the etiology of mild to severe hypersomnolence in depression. We attempted to reconcile findings from both mental health and sleep medicine research, with the aim of identifying potential clinical targets to enhance phenotyping, diagnosis, and treatment strategies.

## Methods

For this narrative review, we first reviewed diagnostic manuals in psychiatry (DSM-5-TR) (5) and in sleep medicine (ICSD-3-TR) (6) and extracted epidemiological and pathophysiological information as well as references used. Then, we searched references in PubMed published between January 1, 1990, and 2024 using the MeSH “Disorders of Excessive Somnolence,” “Depressive Disorder,” and “Bipolar and Related Disorders” (Supplement, section S1). Inclusion/exclusion criteria are detailed in the Supplement, section S2. Briefly, we included only peer-reviewed articles published in French or English. All meta-analyses and systematic reviews relevant to the topic were included. Reference lists of retrieved articles were also searched. For original research reports, no prespecified quality assessment tools were applied; however, studies were prioritized based on several factors, including sample size, study design, and whether findings had been replicated. Additional clinical interpretation is provided based on the authors’ experience.

## Epidemiology

The prevalence of hypersomnia co-occurring with a psychiatric disorder varies greatly with the definitions used. The ICSD-3-TR reports that it accounts for 11% to 19% of PSG/MSLT referrals (6), but up to 25% of patients with a depressive disorder were reported to present with hypersomnolence (2,3). Hypersomnolence may predispose to and perpetuate depression (15, 16, 17, 18, 19), with a co-evolutionary relationship being suggested by associations between the severity of hypersomnolence, depressive symptoms, and functional consequences (20,21).

The sex distribution needs clarification, but hypersomnolence in depression is reported more often by women, who also report more severe symptoms (21, 22, 23, 24). The sex difference is hypothesized to result from a myriad of factors ranging from social roles and cultural norms to different exposure to gonadal hormones (24), namely progesterone, which has hypnotic properties (25, 26, 27, 28). Nonetheless, insomnia remains more prevalent than hypersomnolence in women (29), which suggests interindividual variability in the response to hormones.

The age distribution of hypersomnolence differs from the typical U-shaped pattern of depressive symptoms (30). Hypersomnolence is rare in childhood (31), rises to around 75% in young adults (32), and falls below 50% in older adults with depression (32). Similarly, IH has a mean age of onset of 16 to 21 years, with approximately 30% remission 5.5 years after diagnosis as per a retrospective study (6,33). The distribution of the prevalence of hypersomnolence is also reminiscent of the evening chronotype distribution across life (34). Such circadian delay could contribute to explaining sleep inertia, morning sleepiness, and sleep-onset insomnia in younger adults with depression, as opposed to the early morning awakenings commonly reported by older adults with depression (35). Circadian mechanisms are reviewed in the next section.

The prevalence of hypersomnolence varies by depression subtype, although specific links with EDS, hypersomnia, and sleep inertia are unclear. Hypersomnolence is often associated with both bipolar disorders (euthymic or depressed phases) and certain subtypes of MDD with a bipolar diathesis (atypical or SAD), leading some authors to suggest that it could be a trait marker for bipolar disorder (36). Atypical depression is associated with hypersomnia by definition, as well as hyperphagia and leaden paralysis, and it accounts for approximately 15% to 29% of patients with MDD (37). It is more common in patients with a higher number of depressive symptoms and is more frequent in women than in men (38). Patients with SAD also often present with psychomotor retardation and hyperphagia (5), and 50% to 80% report hypersomnia (6,39,40). Hypersomnolence is also commonly seen in bipolar disorders during both euthmyic and depressive episodes. A meta-analysis found that 30% of patients with bipolar disorder (depressive/euthymic) complained of hypersomnolence, independent of medication status (41). Interestingly, an association was found between EDS and a subsequent hypomanic/manic switch approximately 7 months later, but no such association was found with increased time in bed (42). This differential association emphasizes hypersomnolence’s heterogeneity, suggesting that EDS or excessive time in bed may need separate assessment to link them to depressive symptoms or even subtypes.

## Pathophysiological Models of Hypersomnolence in Depression

Hypersomnolence is heterogeneous and may stem from a myriad of factors. Table 1 summarizes key findings on hypersomnolence etiologies as well as hypotheses that require further assessment.

**Table 1:** Hypothesized Neurohormonal Factors Involved in the Etiology of Hypersomnolence in Depression

Neurobiological Factors	Hypothesized Mechanism
Circadian Dysfunction	Circadian phase delay
	Increased circadian period
	Decreased circadian amplitude
	Reduced light sensitivity
Structural/Functional Anomalies	Hypoperfusion/hypoconnectivity of the medial prefrontal cortex (part of the default mode network)
	Decreased slow wave activity in the supramarginal gyrus
HPA Axis Dysregulation	Decreased secretion of CRH
	Decreased sensitivity to CRH
Altered Monoaminergic Systems	Decreased dopaminergic activity
	Decreased noradrenergic activity
Altered GABA Function	Decreased nighttime GABA_B_ activity
	Increased daytime GABA_A_ activity
Altered Response to Sex Hormones	Increased sleepiness with circulating hormones

CRH, corticotropin-releasing hormone; GABA, gamma-aminobutyric acid; HPA, hypothalamic-pituitary-adrenal.

### Sleep Studies

Ad libitum PSG studies have found that patients with depression who reported hypersomnia showed that total sleep time duration was increased or similar to control groups over 24 hours (43, 44, 45, 46, 47, 48). Accordingly, the ICSD-3-TR reports modestly prolonged sleep in hypersomnia associated with a mental disorder (6). Other marginal findings on the PSG include increased total time in N1 at the expense of a reduction of N3 sleep (47,49,50). To date, no reliable PSG marker has been found to identify hypersomnolence associated with depression. An 8-hour PSG study suggested that those patients had decreased sleep efficiency, increased sleep latency, and increased wake after sleep onset compared with control participants (46). A meta-analysis later showed that patients with psychiatric disorders (mostly depression) and comorbid hypersomnolence have sleep efficiencies comparable to healthy control participants (43). Inconsistencies across studies may stem from interindividual variability in time spent in bed or differences in how rigorously these behaviors were assessed prior to studies.

Using high-density electroencephalography (EEG) during an ad libitum PSG, Plante *et al.* (51) found a similar EEG signature in unmedicated patients with hypersomnolence compared with participants without hypersomnolence, regardless of depression status (52). Specifically, patients with hypersomnolence had less slow wave activity in the bilateral centroparietal area than control participants. Slow wave activity in this region inversely correlated with subjective measures of EDS, offering a potential marker of hypersomnolence. The authors hypothesized that a supramarginal gyrus anomaly might explain this difference because this region was associated with EDS in other disorders, like narcolepsy (52). Those findings echo earlier studies that showed reduced slow wave activity in participants with IH versus control participants (53). Therefore, the mechanisms that underlie hypersomnolence may be independent of the presence of depression (51).

In a longitudinal study of 1287 individuals of the Wisconsin Sleep Cohort, Plante *et al.* (54) found divergent associations between hypersomnolence components and depression. They found an increased odds ratio (OR) for depression in participants who reported EDS (Epworth Sleepiness Scale ≥ 11, OR = 1.56 [95% CI, 1.31–1.86]), but paradoxically, a mean sleep latency <8 minutes on the MSLT was associated with a decreased odds ratio for depression (OR = 0.76 [95% CI, 0.63–0.92]), which could reflect misattribution of fatigue as sleepiness or limitations of the MSLT (54). They also found an association between depression and long self-reported sleep duration (OR = 2.01 [95% CI, 1.49–2.72]), which appeared to be mediated by self-reported insomnia. The authors proposed that in depression, prolonged subjective sleep duration may be linked to excessive time in bed rather than actual sleep time. In another longitudinal study of 1741 individuals of the Penn State Adult Cohort, Fernandez-Mendoza *et al.* (16) found increased sleep-onset latency in patients with comorbid hypersomnolence and depression compared with participants who had only hypersomnolence or only depression. Notably, medication use was not reported in the article. Increased sleep-onset latency suggests hypervigilance, as in insomnia, rather than central hypoarousal, which is typical of hypersomnolence (44). Fragmented nighttime sleep may contribute to daytime sleepiness or, in some cases, a phase delay that manifests with morning sleepiness/sleep drunkenness (6). This pattern may reflect insomnia and fatigue misattributed as hypersomnolence. However, we emphasize that some patients with depression may have hypersomnolence similar to that seen in other central hypersomnolence disorders, as shown by the high-density EEG study of Plante *et al.* (51). Multiple conditions presenting as hypersomnolence in depression may explain MSLT’s limited sensitivity and specificity in that population. A meta-analysis showed that 25% of patients with psychiatric hypersomnolence do in fact demonstrate pathological sleepiness, with a mean sleep latency ˂8 minutes (6,55). Whether such findings are linked to false positives, prior sleep deprivation, or central hypersomnolence remains unclear. Ambulatory EEG over multiple nights may enhance insight into this matter.

### Circadian Studies

**Figure 1:**
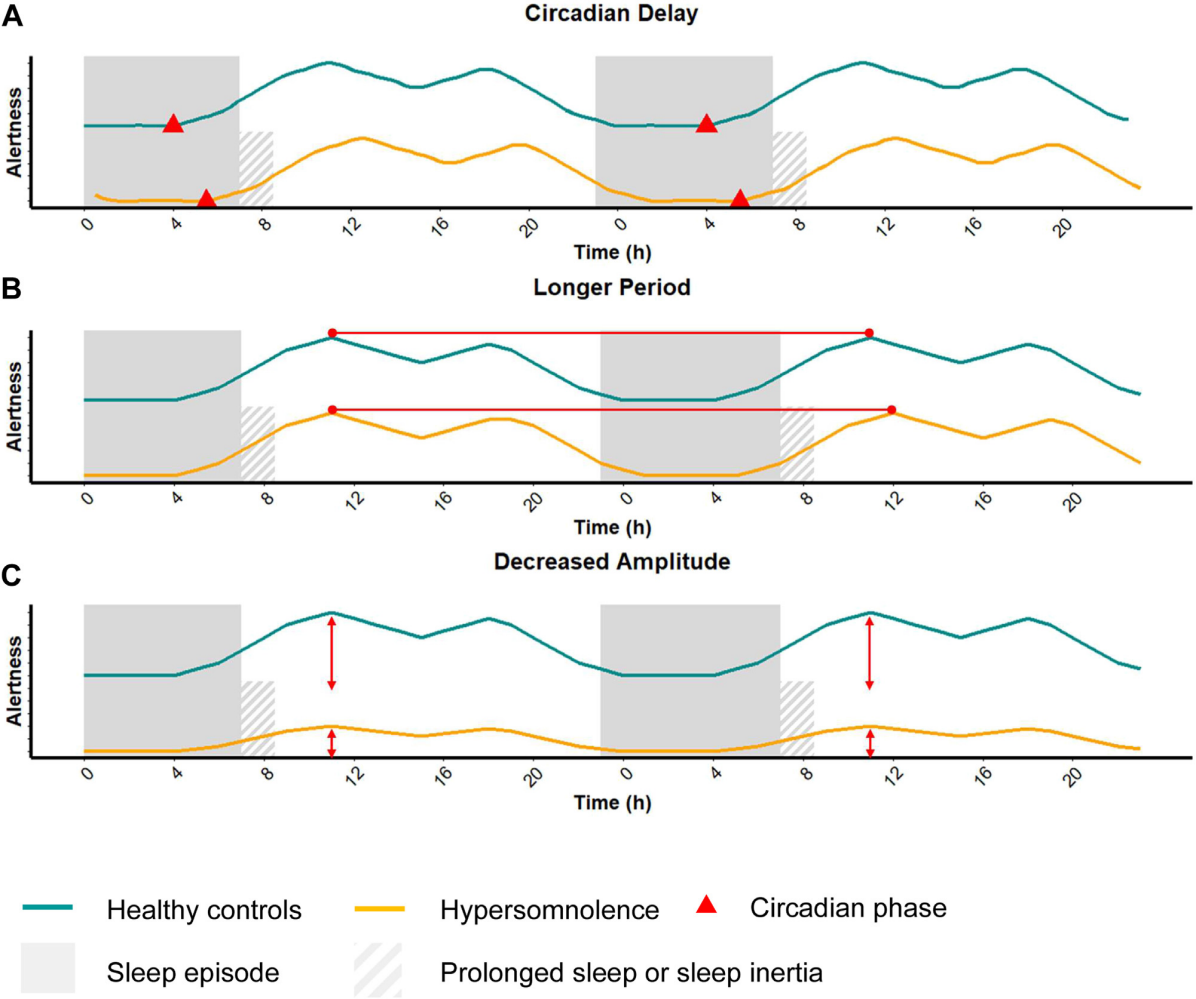
Circadian mechanisms that potentially contribute to hypersomnolence. Various circadian disturbances can co-occur and possibly contribute to hypersomnolence including a delayed phase, an increased period, and a decreased amplitude. All those mechanisms could decrease alertness upon awakening and may prolong the sleep episode or cause sleep inertia.

The circadian system regulates the sleep-wake cycle and may provide insight into the pathophysiology of hypersomnolence in depression, as shown in Figure 1. The circadian system has a period close to 24 hours but needs daily synchronization by time givers or zeitgebers (56). The main zeitgeber is the light-dark cycle. In SAD, the shortening of the photoperiod and the overall reduction in light levels in fall/winter are hypothesized to result in a reduced capacity to properly align with the environment, thus resulting in signals of somnolence at inappropriate times and a complaint of hypersomnolence (57,58). Accordingly, low circadian amplitude and delayed phase are often found in SAD while patients are symptomatic, but whether those changes cause the symptoms or co-occur with them remains unknown (59). Because studies have found antidepressive properties of light therapy at moments it is not expected to significantly correct a delayed phase, the amount of light received may play a greater role in SAD than mistimed light exposure (60). The hypotheses that link a circadian misalignment and hypersomnolence in depression have also been used in bipolar disorders. Although findings have been inconsistent, many studies have found altered circadian rhythms in patients with bipolar disorder, including phase delays or advances, which could result from disturbed sleep patterns and light-dark exposure in mood episodes. Altered circadian rhythms may otherwise be epiphenomenal or contributive to symptoms. Interestingly, increased light sensitivity was found in that population, which may be associated with the symptomatology (61). There appears to be a seasonality to mood symptoms in bipolar disorders, with manic episodes being more common during the spring and depressive episodes being more frequent during fall/winter (62). Adjunctive use of glasses that block blue light was also found to be associated with rapid reduction in manic symptoms in a randomized placebo-controlled trial (63). The specific mechanisms that link circadian abnormalities to hypersomnolence in bipolar disorder are still unclear and lack strong supporting evidence (64).

The literature on circadian rhythms and IH has expanded recently, with some authors suggesting that it may be a circadian rhythm disorder (65). By measuring the circadian period length in peripheral skin fibroblasts of patients with IH, Materna *et al.* (66) found an increased period length compared with control participants. Therefore, it was hypothesized that the longer circadian period might contribute to explaining sleep inertia in that population. This finding must be interpreted cautiously given that 1) period lengths obtained via fibroblasts tend to be higher than when measured in vivo, and 2) the correlation between the circadian period measured with fibroblasts and in vivo via a desynchronization protocol is weak (67,68). Another study of skin fibroblasts showed dampened circadian amplitudes of the expressed clock genes *BMAL1*, *PER1*, and *PER2* in IH compared with healthy control samples (69). Whether these anomalies of the peripheral circadian clock are also present at the central clock remains to be explored (70).

Rach *et al.* (71,72) found a decreased daytime melanopsin-mediated pupil response in patients with IH compared with healthy control participants. Similar results have also been found in MDD (73,74), although the association was not reproduced in milder depressions (75). In a small study, euthymic patients with bipolar disorder (*n* = 9) but not bipolar patients with depression (*n* = 7) had decreased melanopsin-mediated pupil response compared with control participants (*n* = 35) (76). The melanopsin dysfunction could contribute to the symptomatology of hypersomnolence. Light has circadian-independent effects on sleep and alertness, notably through the preoptic circuit (77). Light, via the intrinsically photosensitive retinal ganglion cells, stimulates norepinephrine neurons in the locus coeruleus and inhibits the sleep-promoting ventral lateral preoptic area (78, 79, 80, 81). Decreased melanopsin sensitivity can be conceptualized as decreased light exposure, thus reducing alertness and wakefulness signals promoted by light (82,83). Decreased melanopsin sensitivity could also interfere with the entrainment of the circadian system to the light-dark cycle (84). Unfortunately, circadian phase was not measured in the study of Rach *et al.* (71), but earlier studies reported delayed circadian phase of cortisol and melatonin rhythms in patients with IH compared with control patients (85), with a later chronotype reported in another study (86). The melanopsin-mediated pupil response was not correlated with depressive symptoms in patients with IH, possibly because depressive symptoms were low in participants. The concept of light-related vulnerability to sleepiness may help in the classification of hypersomnolence subtypes, although it is based on limited evidence and needs to be tested in future studies (71).

### Neuroimaging

No systematic neuroimaging study has been conducted with patients with a depressive disorder and hypersomnolence, and few studies have been conducted with patients with IH. A study using functional magnetic resonance imaging found lower functional connectivity within the anterior default mode network (DMN) (medial prefrontal cortex) in patients with IH than control participants during wakefulness (87). The DMN is of interest in presentation of hypersomnolence given that it is closely associated with the maintenance of alertness and is dynamically modulated during sleep (88,89). Lower functional connectivity of the DMN during wakefulness is reminiscent of the DMN decoupling that occurs during sleep (88). Interestingly, this anomaly correlated with reported daytime sleepiness in patients with IH (87). In parallel, greater volume and cortical thickness were found in the precuneus (posterior DMN) of patients with IH. Because cortical thickness of the posterior DMN was inversely correlated with the cortical thickness of the anterior DMN, the authors suggested that it might reflect compensatory changes to lower functional connectivity in IH. Likewise in depression, a study showed decreased anterior connectivity in the DMN, with increased posterior DMN activity (90). Such an anomaly could contribute to explaining hypersomnolence in some individuals with depression, which remains to be assessed systematically. Other studies found no DMN changes (91) or DMN changes in the opposite direction, which emphasizes the heterogeneity of depressive symptoms and the need to refine studied populations (92). Some participants with IH also underwent a single-photon emission computerized tomography scan (93). In addition to the lower functional connectivity in the medial prefrontal cortex, lower regional cerebral blood flow was found in that area. This anomaly was also associated with higher daytime sleepiness and differed from previous findings of studies that assessed the effects of sleep deprivation in healthy control participants (94). Therefore, those findings may be specific to IH rather than a more generalizable marker of individuals experiencing sleepiness (93). Studies are needed in depression to confirm those findings, especially because the medial prefrontal cortex dysfunction is associated with depressive disorders and may be a treatment target for neuromodulation (95,96).

### Dysregulation of the Stress Axis

The stress system is hypothesized to be deregulated in MDD. In euthymic individuals, the subgenual prefrontal cortex inhibits the amygdala, the hypothalamic-pituitary-adrenal (HPA) axis, and the sympathomedullary system (97). Under stress, this inhibition is partially lifted, which activates these systems. This promotes physiological hypervigilance and anxiety, while decreasing sleep and appetite (97). In melancholic depression, the subgenual prefrontal cortex is both functionally and structurally impaired, with a size reduction of about 40% (98,99). The loss of inhibition of the stress system results in the activation of the amygdala, HPA axis, and the sympathomedullary system, which translates into increased levels of anxiety, early morning awakening, and anorexia (32). In contrast, the hypersomnia and hyperphagia observed in atypical depression were suggested to result from an exaggerated inhibition of the stress response (100). The mechanisms that underlie this process remain to be elucidated. One hypothesis suggests that chronic hyperactivity, as seen in melancholic depression, may inhibit the HPA axis through a negative feedback loop, possibly by reducing corticotropin-releasing hormone secretion or sensitivity to it (99). Low corticotropin-releasing hormone concentrations in cerebrospinal fluid have been reported, accompanied by low plasma adrenocorticotropic hormone levels (101). The reduction of hypersomnia in patients with atypical depression by St. John’s Wort (102), an HPA axis activator (103,104), also favors this model, but the overall level of evidence remains weak.

A similar hypothesis has been proposed for the symptomatology observed in SAD. The overlapping hallmark symptoms between SAD and atypical depression may suggest the involvement of similar pathways (50), although the sole involvement of the HPA axis is not sufficient to explain the seasonality of the phenomenon. An alteration of the circadian system across the seasons could interact with the HPA axis, but that remains to be demonstrated.

### Neurotransmission

Preclinical models support the implication of norepinephrine and dopamine in hypersomnolence (105), which is consistent with pharmacological studies that have demonstrated improvement of sleepiness with noradrenaline and dopamine reuptake inhibitors (106, 107, 108). Furthermore, low levels of norepinephrine in cerebrospinal fluid correlated with EDS in participants with central disorders of hypersomnolence (109). Conversely, sodium oxybate in IH is mainly hypothesized to improve nighttime sleep via its action on GABA_B_ (gamma-aminobutyric acid B) receptors located on the thalamocortical, dopaminergic, and noradrenergic neurons (110). Taken together, the pharmacological evidence from the study of hypersomnolence suggests involvement of the dopaminergic and noradrenergic system—both while awake and asleep. An anomaly of these circuits may explain the benefits of increasing those monoamines during the daytime (with noradrenaline and dopamine reuptake inhibitors or similar molecules) and inhibiting them at night (with sodium oxybate). This simplified model emphasizes that most first-line treatments for depression (e.g., selective serotonin reuptake inhibitors) are insufficient to address hypersomnolence in depression (111).

There is also limited evidence for the involvement of GABA_A_ receptors in hypersomnolence. Although an endogenous hypnotic factor that activates GABA_A_ receptors was identified (112), this finding has not been reproduced by another group (113). Small trials of negative allosteric modulators of GABA_A_ receptors, including clarithromycin (114) and flumazenil (115), have shown some benefits in treating hypersomnolence, but sample size and study designs limit the generalizability of those findings (107).

In IH, normal cerebrospinal fluid levels of other key neurotransmitters were found, including orexin, histamine, and tele-methylhistamine (116, 117, 118).

### Psychological Factors of Hypersomnolence in Depression

**Figure 2:**
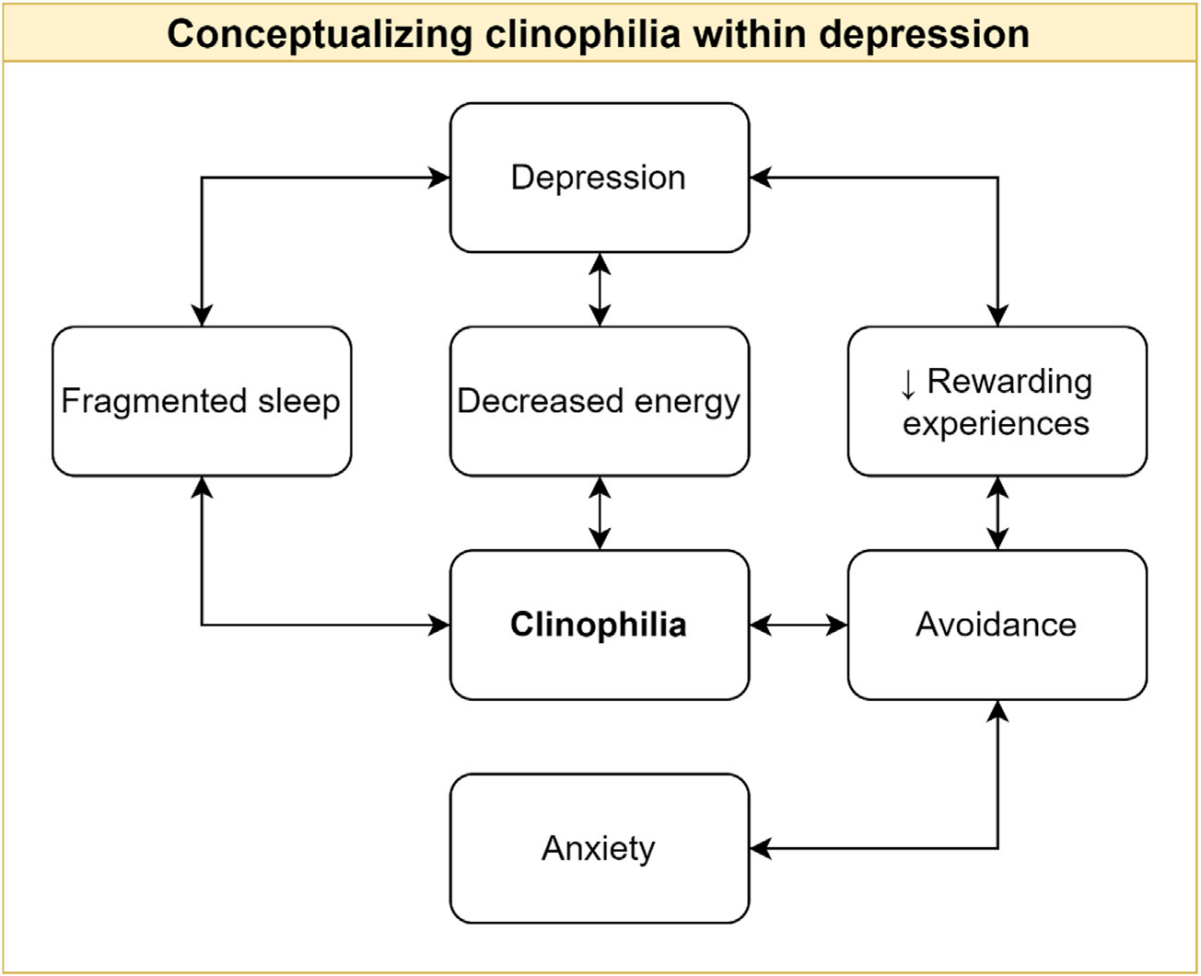
Contributing factors to clinophilia in depression, a common mimicker of hypersomnolence. This figure depicts a simplified model of the complex interplay between depression, anxiety, and clinophilia and is largely based on the cognitive behavioral model of depression. Depression is associated with fragmented sleep and decreased energy, which may lead to excessive time in bed in an attempt to compensate for those incapacitating symptoms. Depression is also characterized by a decrease in rewarding experiences that may prevent individuals with depression from engaging in usual daily activities/seeking new experiences. The resulting avoidance of exposure to the outside world may lead to clinophilia. In parallel, anxiety (which is often comorbid with depression) often results in maladaptive avoidance and possibly to clinophilia.

Psychological factors have also been identified as contributing to the presentation of hypersomnolence in depression. The ICSD-3-TR describes the presence of cognitive distortions and maladaptive behaviors in hypersomnia associated with a psychiatric disorder (6). Namely, it has been reported that “patients may be intensely focused on their hypersomnolence” and that some spend an excessive amount of time in bed without sleeping, a phenomenon called clinophilia (6). The mechanisms that lead to clinophilia in depression are presented in Figure 2.

It has even been suggested that large discrepancies between total sleep time and time in bed distinguish depression from IH (44). Some studies using actigraphy found that patients with psychiatric disorders showed minimal movement for >12 hours at home but had a normal sleep duration in the laboratory, thus suggesting a long period of time spent in bed at home (44,45). An explanation for the inconsistency between subjective and objective measures of sleepiness in depression is that individuals with mood disorders may confound fatigue with sleepiness. This interpretation is plausible given negative cognitive biases and dysfunctional beliefs often observed in mood disorders (54,119). Parker *et al.* hypothesized that the complaint of hypersomnolence in depression might reflect a dysfunctional coping strategy rather than a depressive symptom per se, because hypersomnolence was reported independently of depressive subtypes in their study (32).

Evidence also exists regarding the presence of a positive sleep state misperception (subjective sleep duration > objective sleep duration) in patients with depression who report hypersomnia. In a relatively large study (*N* = 147), participants with SAD reported unhelpful beliefs about sleep more often than control participants (120). Specifically, patients with SAD and hypersomnia reported higher scores on a measure of misbeliefs around sleep, thus suggesting that cognitive distortions may contribute to the subjective experience of hypersomnolence.

## Limitations

This review has several limitations. First, it was not prospectively registered, and the broad scope and poorly defined hypersomnolence phenotypes in depression made a systematic review impractical. Second, much of the evidence comes from small, nonreplicated studies. Despite these limitations, addressing hypersomnolence in depression is essential because it exposes significant gaps in clinical knowledge and practice.

## Conclusions

This review highlights several potential mechanisms underlying hypersomnolence, each with implications for diagnosis and treatment. Reduced daytime dopaminergic and noradrenergic activity may contribute to EDS, and accordingly treatments aimed at enhancing these neurotransmitters can sometimes alleviate symptoms. Nighttime GABA hypoactivation may disrupt sleep continuity, pointing to potential benefits from interventions that target GABAergic pathways such as oxybates. Sleep improvement could lead to long-term mood enhancement, although neuropsychiatric side effects of the medication must be considered. Expanding on the role of GABA in MDD, evidence indicates that reduced GABA levels and impaired inhibitory function contribute to MDD neurobiology (121). This GABAergic disruption may lead to the alterations in DMN connectivity that have been observed in MDD (122). GABA and DMN anomalies have similarly been reported in IH, although the findings are still preliminary. Based on these associations, we hypothesize that disrupted GABA-DMN interactions could underlie hypersomnolence in MDD, which warrants further investigation. Exploration is also warranted into whether neuromodulation techniques that target the medial prefrontal cortex, where hypoperfusion and hypoconnectivity have been observed, could alleviate both hypersomnolence and mood disturbances in depression (9). Circadian dysregulation, such as a longer period and light hyposensitivity, suggests that blue-enriched phototherapy or dawn simulators may help manage hypersomnolence in depression, particularly when circadian disturbances are prominent. Objective measurements, such as circadian phase and period assessments (e.g., fibroblast-derived circadian period) or light sensitivity tests (e.g., melatonin suppression test, melanopsin-mediated pupil response), could also provide more precise biomarkers for identifying hypersomnolence disorders in depression. Clinicians and researchers must exercise caution in distinguishing hypersomnolence from psychophysiological hyperarousal because the latter can result in fragmented sleep and EDS, mimicking hypersomnolence. Ambulatory PSG may assist in distinguishing those phenotypes. Maladaptive behaviors like clinophilia can also contribute to a clinical complaint of hypersomnolence, which underscores the importance of developing cognitive behavioral therapy tailored to hypersomnolence.

EDS, hypersomnia, and sleep inertia may benefit from being studied separately when concomitant to psychiatric disorders, as emphasized by their differential association with prognosis in bipolar disorder. Other clinical features of hypersomnolence may also help refine studies by increasing the homogeneity of samples. For example, recent evidence suggests that patients with IH may gain from being further phenotyped on whether naps are refreshing or not. Patients with unrefreshing naps were found to have less fragmented sleep, which may be a marker of weaker arousal drive (123). The use of this clinical phenotyping could be explored in depression, and it could help identify underlying mechanisms and potentially allow for tailored treatment.
