# Supplementary material for: Pathophysiological Models of Hypersomnolence Associated With Depression
Source: Biol Psychiatry Glob Open Sci. 2024 Dec 26;5(2):100445. doi: 10.1016/j.bpsgos.2024.100445 (PMC11810709; doi:10.1016/j.bpsgos.2024.100445)
Supplement: Supplement Text [file mmc1.pdf]

SUPPLEMENTARY INFORMATION

Pathophysiological Models of Hypersomnolence Associated With Depression

Moderie and Boivin

S1. Research strategy in Pubmed

(((Disorders of Excessive Somnolence[MeSH Terms]) OR (Hypersomnia[MeSH Terms]) OR (Excessive Daytime Sleepiness))

AND ((Depressive Disorder[MeSH Terms]) OR (Bipolar and Related Disorders[MeSH Terms])))

AND ("1990/01/01"[Date - Publication] : "2024/01/01"[Date - Publication])

AND (English[Language] OR French[Language])

S2. Inclusion and exclusion criteria for reviewed studies

This review is a narrative synthesis rather than a systematic review and was therefore not preregistered. No pre-specified quality assessment tools were applied; however, studies were prioritized based on several factors, including sample size, study design, and replication of findings, to provide a comprehensive yet discerning overview of the existing evidence.

Inclusion Criteria:

1. Language:

- Articles in English or French.

2. Peer Review Status:

- Only peer-reviewed publications were included.

3. Publication Type:

- Meta-analyses and systematic reviews retrieved were all included.
- Systematic reviews, narrative reviews, and original research articles included if relevant.
- Studies on treatment included if they provided insights into pathophysiology; other treatment-focused studies reviewed separately.

4. Study Population:

- Primarily adult populations; adolescent studies included if relevant to early-onset hypersomnolence in mood disorders.

5. Diagnostic Relevance:

- Studies applying DSM and ICSD diagnostic criteria.

6. Timeframe:

- Publications from January 1, 1990, to 2024.

7. Study Rigor and Relevance:

- Original studies with larger sample sizes and replicated findings were prioritized.

Exclusion Criteria:

Studies not meeting the above inclusion criteria were excluded, in addition :

1. Peer Review Status*:*

- Non-peer-reviewed articles were excluded.

2. Publication Type*:*

- Studies reporting preliminary results were excluded if the final analysis was available.
- Case reports, opinion pieces, and conference abstracts were excluded unless providing unique insight into the pathophysiology of hypersomnolence in mood disorders.
